# Exploring therapeutic communication in managing chronic non-communicable diseases: a mixed-method study in Ghana

**DOI:** 10.1186/s13690-024-01263-y

**Published:** 2024-03-14

**Authors:** Ethel Rhodaline Ameworwor, Hubert Amu, Robert Kokou Dowou, Gideon Kye-Duodu, Selasi Amu, Luchuo Engelbert Bain

**Affiliations:** 1 Department of Epidemiology and Biostatistics, Fred Newton Binka School of Public Health, University of Health and Allied Science, Hohoe, Ghana; 2 Department of Population and Behavioural Sciences, Fred Newton Binka School of Public Health, University of Health and Allied Science, Hohoe, Ghana; 3 Department of Psychology, Faculty of Humanities, University of Johannesburg, Johannesburg, South Africa; 4 Department of midwifery, School of Nursing and Midwifery, University of Health and Allied School, Ho, Ghana

**Keywords:** Therapeutic communication, Chronic non-communicable diseases (CNCDs), Ghana, Facilitators, barriers

## Abstract

**Background:**

Globally, the burden of chronic non-communicable diseases is increasing rapidly and approximately one in three of all adults suffer from multiple chronic conditions. Therapeutic communication plays a crucial role in achieving curative, preventive, and promotive goals regarding chronic disease management. We examined therapeutic communication between health professionals and patients with chronic non-communicable diseases at the Ho Teaching Hospital.

**Methods:**

We adopted a concurrent mixed-methods approach. The quantitative aspect of the study was descriptive while the qualitative was explanatory. The quantitative study was conducted among 250 patients. The qualitative data was collected among eight health professionals. A stratified sampling and simple random sampling methods were used to recruit patients for the quantitative survey while purposive and convenient sampling was used for the qualitative aspect of the study. The quantitative data was collected using a semi-structured questionnaire while the qualitative data was collected using an in-depth interview guide. The quantitative data were analyzed using STATA v17 and the qualitative data were analyzed thematically using Atlas ti. The major themes that emerged were, therapeutic communication practices, barriers to therapeutic communication and possible solutions to good therapeutic communication.

**Results:**

We found that 37% patients were 60 years and above with 53.2% being females. It was noted that 36.4% of patients have had tertiary-level education. We found that 59.2% of patients reported having good therapeutic communication with health professionals. We, however, noted that male participants were 92% less likely to practice good therapeutic communication compared with females (aOR = 0.92,95% Cl = 0.46–1.84**)**. Health professionals’ activities to ensure good therapeutic practices included their capacity to listen, build rapport with their patients, and clarify information. We found that the major facilitators of good therapeutic communication included trust in the health professionals (90.4%), conducive environment (93.2%), using simple and plain language by health professionals (92.0%) We found that there are myriad of barriers that impede communication process. This included language, health professionals’ inability to break terminologies, and the unconscious state of patients.

**Conclusions:**

The study revealed that there was good therapeutic communication between health professionals and patients with CNCDs. Nevertheless, it was also identified that ineffective therapeutic communication between health professionals and their patients due to barriers like language could lead to dissatisfaction with care, misdiagnosis, and noncompliance to treatment regimen. For Ghana as a country to achieve SDG target 3.4 by reducing mortality due to NCDs and improve wellbeing of patients by 2030, it will be imperative on Ghana Health Service to design communication strategy training for health professionals that could help improve therapeutic communication between patients and health professionals.

**Table:** 

**Text box 1. Contributions to the literature**
1. The study emphasizes the need for better communication strategies to bridge the language gap and simplify medical jargon for improved patient understanding.
2. The study’s emphasis on the facilitators of good therapeutic communication offers practical insights for healthcare practitioners and policymakers to improve patient care and outcomes.
3. Active engagement of patients in good therapeutic communication results in enhanced patient self-management of their condition(s).

## Background

Chronic non-communicable diseases (CNCDs) are the main cause of death and disability worldwide and their management requires a long-term health plan [1]. The communication between a patient and their health professional is the foundation of an effective therapeutic process in managing CNCDs [2]. Therapeutic communication plays a crucial role in achieving curative, preventive and promotive goals regarding chronic disease management. Globally, approximately one in three of all adults suffer from multiple chronic conditions [3]. There is an increasing burden of non-communicable diseases (NCDs) across the world and particularly in low and middle-income countries (LMIC) as they go through the epidemiological transition from infectious to chronic diseases that accompanies economic development [4]. Even though there is a lack of reliable and accurate data on the prevalence of CNCDs in Sub-Saharan Africa (SSA), CNCDs are expected to exceed infectious diseases as major causes of morbidity and mortality by the year 2035 [5].

In Ghana, the burden of CNCDs, accompanied by their mortalities among the population has achieved epidemic proportions in the past decade [6]. Patient-Centered Care [PCC] has moved to the center stage on issues of healthcare delivery and has received enormous attention as an effective approach to delivering quality healthcare that improves patients’ experience of care [7]. Therapeutic communication refers to the exchange of information between healthcare providers, patients, and caregivers with the aim of developing a relationship that benefits the well-being of the individual [8]. Effective communication among clinicians, patients, and their families is essential to quality health care and plays an important role in improving patients’ health [9]. Ineffective communication serves as an obstacle in the provision of standard services. Health provider-patient communication is an inseparable part of the patients’ care in every health setting; it is one of the factors that determine the quality of care.

The Sustainable Development Goal (SDG) three aims at good health and well-being for all at all ages, hence it is a necessity for all persons with chronic conditions to have access to effective and efficient healthcare through therapeutic communication for this goal to be achieved. Employing good therapeutic communication skills aid to assess patients’ needs and provides them with appropriate physical care, emotional support, knowledge transfer, and exchange of information [10]. Previous studies conducted in Ghana focused generally on communication between nurses and patients with no study on good therapeutic communication between health professionals and people with CNCDs [11–15]. The difficulty in locating peer-reviewed studies in Ghana at large and the study area, informed the need for the current study which sought to explore therapeutic communication between health providers and patients who have complex and chronic comorbid conditions at the Ho Teaching Hospital (HTH). This study examined therapeutic communication between health professionals and patients with CNCDs. The findings could be important in contributing towards the policy aspects of improving the management of CNCDs in Ghana and beyond. It can be through the development of a communication strategy training program for healthcare professionals.

## Methods

The consolidated criteria for reporting qualitative research (COREQ) and strengthening the reporting of observational studies in Epidemiology (STROBE) were adopted in reporting the qualitative and quantitative facets of the study respectively.

### Study setting

Our study was conducted at the Ho Teaching Hospital which is the main referral hospital in the Volta Region in Ghana. The Hospital has a total of 306-bed capacity. The bed capacity ensures that a considerable number of patients can be accommodated for treatment and care. It is the main referral facility in the Volta Region. The hospital employs a dedicated team of healthcare professionals, including medical doctors, nurses and midwives, laboratory technicians, pharmacists, administrative staff and all other healthcare professionals. The hospital provides essential services in the following departments: Obstetrics and Gynaecology, Paediatrics, Surgery, Medicine, Psychiatry, and Dentistry. It also has specialist clinics which include Sickle Cell, Hypertension, Diabetes, Infertility, Cerebral Palsy, Uro-Gynaecology, Dental, Physiotherapy, Anaesthesia, and Pain Clinic, Anti-Retroviral Therapy, Eye Clinic. HTH experiences a significant patient load with a considerable number of patients seeking healthcare services on a daily basis [16]..

### Study design

The study adopted a concurrent mixed-method approach (qualitative and quantitative methods) to help provide a comprehensive understanding of the situation. The quantitative aspect of the study was a descriptive cross-sectional design while the qualitative aspect was exploratory in nature. We used an exploratory qualitative design to gather in-depth information on health professional’s therapeutic communication practices and barriers. A descriptive quantitative design enabled us to obtain evidence on patients’ therapeutic communication practices, facilitators, and barriers.

### Sample size determination

The quantitative sample size was determined using Cochrane’s single proportion formula:

$$ n=\frac{{z}^{2}p\left(1-p\right)}{{d}^{2}}$$

Where,

n = sample size.

$$ {Z}_{ }^{2}$$= reliability co-efficient

*p* = prevalence hypertension.

𝑑^2^ = margin of error.

The reliability co-efficient used was 1.96 as per a confidence level of 95% with the margin of error as 0.05. In a study conducted in Volta region, the prevalence of hypertension in Ho was found to be 18.70%, which was used in determining the sample size [17].

$$ \text{S}\text{a}\text{m}\text{p}\text{l}\text{e}\,\text{s}\text{i}\text{z}\text{e} \left(\text{n}\right) =\frac{{1.96}^{2}\text{X} 0.187 (1-0.187)}{({0.05)}^{2}}=233.617$$

This was approximated to 234.

To account for the non-response rate, 5% of the calculated sample size was added to the sample size. Hence 5% of non-response rate = 0.05 × 234 = 11.7.

Therefore, the final sample size required for this study was = 246. However, to take care of incomplete data during cleaning we decided to use 250 which is the maximum sample size with our believe that a larger sample size increases the precision of the data.

For the qualitative study, a sample of eight (8) participants were interviewed which included four (4) nurses, and four (4) physiotherapists).

### Population and sampling

People with CNCDs who have used HTH services for their ailments were the study’s primary target. The quantitative sample targeted any patient living with hypertension, diabetes, and stroke who accessed treatment services from HTH whereas health professionals who directly manage CNCDs were equally targeted. The qualitative sample consisted of nurses and physiotherapist who were at post for more than six months.

### Sampling

#### Quantitative sampling procedure

A stratified sampling and simple random sampling methods were used to recruit participant. This method was employed to obtain maximum variation sampling for the various disease types. A stratified random sampling method was used to put all three conditions into three [3] strata and this was done to help achieve maximum variation sampling. These strata consisted of stroke, hypertensive and diabetic patients. Simple random selection was finally done to select participate from each stratum. A lottery method was employed with participants who selected yes and consented. This resulted in the sampling of 250 patients for all 3 disease conditions of which they all participated.

#### Inclusion and exclusion criteria

This study included patients who were diagnosed with hypertension, diabetes, or stroke and attending weekly check-ups. Also, health professionals who care for these patients and have been working for more than 6 months were included. However, we excluded patients who were diagnosed with CNCDs other than hypertension, diabetes, or stroke. Also, patients who were diagnosed with hypertension, diabetes, or stroke but were very ill and were not able to clearly communicate with the research team were also excluded. Health professionals who were not available during the time of data collection were excluded from the study. **Qualitative sampling procedure**.

Purposive and accidental sampling procedures were used in recruiting study health professionals. The participants were purposively selected to participate in the study based on characteristics that were of interest. Moon [18] stated that the logic and power of purposeful sampling lie in selecting information-rich cases for in-depth study. A total of 8 health professionals participated of which the size was based purely on data saturation.

### Data collection

A questionnaire was the data collection instrument used to collect quantitative data from the patients. A pre-tested semi-structured questionnaire was adopted to collect data from respondents through Kobo collect application. Face-to-face administration of the questionnaire was done. To protect the privacy of the interviewees, the interviews were conducted from a secluded area so to ensure that third parties could not hear what is being discussed. All interviews were conducted in English and Ewe. Duration for the interviews ranged from 15 to 25 min. The reliability Cronbach alpha test for therapeutic communication practices, facilitators of therapeutic communication, and barriers to therapeutic communication were reported as 0.67, 0.88, and 0.67 respectively.

The qualitative data were collected through an in-depth interview guide that served as the data collection instrument to gather in-depth information from participants. The interview guide allowed for further probing to explore participant contribution into details. An in-depth interview guide was adopted to enhance the investigation of experiences and views on therapeutic communication between health professionals and patients with CNCDs. All interviews with the health professionals were administered face-to-face and were conducted in English. The duration for the interviews ranged from 30 to 35 min. Interviews were recorded and written to avoid bias after which the recordings were transcribed verbatim. The handwritten note and the audio recordings were to help ensure interviews were not halted due to equipment malfunction. We recognise the importance of rigor and trustworthiness in qualitative research, we worked to ensure confirmability and transferability. The study findings were transferable and confirmable due to the detailed description of the study circumstances and techniques. After each interview, field notes were taken and referred to during the analysis, which included the participants’ nonverbal indications, worries, and interviewers’ reflections. Also, the instrument and the interview guides were given to experts in the field of qualitative study for them to peruse in detail the worth of the questions [19]. We also carried out pretest interviews among patients with NCDs in a different health facility (Volta regional Hospital). Patient recruitment for this project were through a key informant, a knowledgeable nurse at the healthcare facility. The nurse informed patients about the research, built trust, and those willing to participate were enrolled after their consultations. The project extended over a period of four months (1st July– 22nd October 2022).

### Study variable

#### Dependent variable

The dependent variables for this study are therapeutic communication practices, facilitators of therapeutic communication, and barriers to therapeutic communication. The questions were formulated based on previous published studies. The questions were in four Likert scale ranging from strongly agree, agree, strongly disagree, and disagree. Patients were asked to tick one of the responses in relation to the question been asked.

#### Independent variable

The independent or explanatory variable for this study was socio demographics. The individual factors included age, sex, religion, ethnicity, occupation, marital status, the mode of healthcare financing, the number of persons living in a household and the CNCD diagnosed.

### Data analysis

The quantitative data was exported into excel for data cleaning and was imported into STATA v17 for analysis. To ensure the quality of the data entered, a double entry was done to address discrepancies during data entry. The quantitative data were analysed using descriptive and inferential statistics. To establish the associations between variables, Chi-square and logistic regression with *p*-values of less than 0.05 were considered statistically significant at a 95% confidence interval. To generate the practice score, the row total command was used in Stata for all questions measuring practice. Using the median as reference point, anything above it was rendered good while those below were bad. The median was used as a reference because it is not influenced by any outlier or the skewedness of the data. Tables were employed to present the results.

For qualitative, thematic analysis was conducted. Data collected were transcribed, proofread, and prepared for analysis by combining all the transcripts into one Microsoft Word file. To minimise errors, transcribed interviews were compared with notes taken during interviews and proofread while listening to the audio recordings. Themes and quotes were developed using ATLAS.ti. V.7.5. All the recorded interviews, as well as notes that were taken during the interview, were transcribed verbatim. Familiarization with the data was done to take note of key ideas and recurrent themes. Coding was done based on the research objectives as well as themes that emerged from the data. We employed the concurrent mixed methods research design to enable broad and detailed account on therapeutic communication between health professionals and patient with chronic non-communicable diseases. This method helps to provide both triangulation and complementarity of data.

## Results

### Socio-demographic characteristics of patients

Table 1 presents the socio-demographic characteristics of patients. This was quantitative in nature with a total of 250 patients being recruited. About 37% were 60 years and above. Females constituted 53.2% and 36.4% had tertiary-level education. The predominant occupation was civil service (28.8%). The majority (93.3%) identified as Christians. Most of them were married (66.8%) and were Ewes (65.6%). About 88% of respondents also indicated that including themselves, they were 5 in a household while 50.8% reported they self-handedly finance their healthcare. The results show that approximately 27.6% of the respondent reported having been diagnosed with hypertension while 21.6% reported living with both hypertension and diabetes.

**Table 1:** Socio-demographic characteristics of CNCDs patients at HTH

Variable	Frequency[*N* = 250]	Percent [%]
**Age**		
20–29	13	5.2
30–39	32	12.8
40–49	38	15.2
50–59	75	30.0
60+	92	36.8
**Sex**		
Male	117	46.8
Female	133	53.2
**Level of education**		
No formal education	28	11.2
Primary	22	8.8
Junior high school	45	18.0
Secondary	64	25.6
Tertiary	91	36.4
**Main occupation**		
Civil servant	72	28.8
Farmer	28	11.2
Trader	46	18.4
Unemployed	52	20.8
Artisan	52	20.8
**Religion**		
African Traditional	8	3.2
Christianity	234	93.6
Islam	8	3.2
**Marital status**		
Never married	25	10.0
Married	167	66.8
Divorced	31	12.4
Widowed	27	10.8
**Ethnicity**		
Akan	35	14.0
Ewe	164	65.6
Ga/Dangme	11	4.4
Guan	33	13.2
Mole-Dagbani	7	2.8
**Persons in a household**		
1–5	220	88.0
6–10	30	12.0
**Health financing**		
Self	127	50.8
NHIS	47	18.8
Family support	76	30.4
**Diagnosed CNCD**		
Diabetes	63	25.2
Hypertension	69	27.6
Stroke	64	25.6
Co-morbid (Diabetes, Hypertension)	54	21.6

Table 2 presents the socio-demographic characteristics of health professionals. It was found that 75% were males while the majority were aged between 25 and 35 years. All the health professionals had tertiary education, with 100% attaining the 1st degree. It was noted that 50% of health professionals were married. Christianity was the most dominant religion among professionals (100%). Half of the health professionals were Ewes. Health professionals interviewed from the hospitals were general nurses (25.0%), specialized nurses (25.0), and physiotherapists (50.0%). It was found that 25% had also worked for 1–2 years while 75% did so for 3–5 years.

**Table 2:** Socio-demographic characteristics of health professionals at HTH

Variable	Frequency (8)	Percentage (%)
**Sex**		
Male	6	75.0
Female	2	25.0
**Age**		
20–29	4	50.0
30–35	4	50.0
**Duration of practice**		
1-2years	2	25.0
3-5years	6	75.0
**Qualification obtained**		
Degree	8	100.0
**Ethnicity**		
Ewe	4	50.0
Akan	3	37.5
Ga	1	12.5
**Unit of work**		
OPD (General nurse)	2	25.0
Cardio (Specialist)	2	25.0
Physiotherapy	4	50.0
**Marital status**		
Never married	4	50.0
Married	4	50.0
**Religion**		
Christianity	8	100.0

### Therapeutic communication practices

Table 3 presents the therapeutic communication practices for CNCD patients at HTH. Evidence from both the quantitative and the qualitative sections of our study indicate that there were good therapeutic communication practices between health professionals and their patients. First to the quantitative findings, the results indicated that the general practices taken in place to have effective therapeutic communication between health workers and patients are good. We found that 59.2% of patients reported having good therapeutic communication with health professionals. A warm welcome is a gateway to effective communication and 239 patients, representing 95.6% of the respondents agreed to this. Time matters when it comes to communication because it allows patients and health workers to express themselves for clear understanding. From the study, 80% of patients agree adequate time is allocated for communication. We found that 752% of patients reported being motivated and sympathized with by health professionals.

**Table 3:** Therapeutic communication practices for CNCD patients at HTH

Variable	Frequency [250]	Percent [%]
**I am warmly welcomed to the health facility anytime I visit**		
Disagree	11	4.4
Agree	239	95.6
**I am well-educated about my health conditions**		
Disagree	74	29.6
Agree	176	70.4
**I am given adequate time for communication and interactions**		
Disagree	50	20.0
Agree	200	80.0
**The health professionals treat me with politeness**		
Disagree	29	11.6
Agree	221	88.4
**They are calm to always give me help when it’s required**		
Disagree	38	15.2
Agree	212	84.8
**I am motivated and encouraged to open up to be provided with the best healthcare needed**		
Disagree	62	24.8
Agree	188	75.2
**When I am leaving the health facility, farewell and words of encouragement are said to me**		
Disagree	129	51.6
Agree	121	48.4
**Generally, how do you feel before leaving the facility?**		
Good	161	35.6
Bad	89	64.4
**General therapeutic communication practices**		
Good	148	59.2
Bad	102	40.8

### Qualitative results

Table 4 presents the themes and sub-themes from our analysis. Three major themes with six sub-themes emerged from the data. These were general management practices by health professionals, barriers faced by health professional and patients with regard to therapeutic communication and some solutions to help improve effective therapeutic communication.

**Table 4:** Thematic table: insights from health professionals at HTH

Main theme	Sub-theme	Code
Practices	General communication practices	• Rapport building • Communicating in a common language • Engaging patient • Serene environment • Teaching to equip patient • Someone to interpret • Giving basic understanding
Barriers	Health system related	• Staff workload • Inadequate logistics • Inability to break terminologies • Lack of monitoring • Patient information • Language
	Patient-related	• Lack of cell phone • Lack of privacy • Finance • Network • Patient status
Solutions	Government	• Improve staff-patient telecommunication • Employ translator • Developing a monitoring system • Finance Provision of equipment
	Facility	• Developing general procedures • Employ the usage of a diagram • Engage caregivers • Deploy multilingual staff • Provision of privacy • Break terminologies
	Patient	• Adherence

### Therapeutic communication practices by health professionals

In line with the qualitative findings, Table 4 shows practices by health professionals in ensuring good therapeutic communication with CNCD patients. Practices regarding therapeutic communication among CNCDs were analyzed by health professionals. It focused on the roles that health professionals play to ensure effective therapeutic communication in the management of CNCDs. The practices ensure that health professionals are able to determine the form of intervention to put in place to improve the health of the patient. The following quotes summarize their responses:

*When you come here, we welcome you here, introduce myself, I am a physiotherapist here, I will be seeing you today. It is a good practice to do every time you meet a patient.*

- Physiotherapist, Male, 35years.

*Usually, when a patient is referred to the unit for the first time, we orient them to the unit environment, what they are supposed to expect at the end of the service and if you have any concerns, we also address them. Show them the way forward.*

- Nurse, Male, 30 years.

Education of patients was also noted by health professionals as one of the practices they carry out as part of their management practices. All eight respondents stated that to practice good therapeutic communication, it was necessary to educate the patient very well. The comments below illustrate their points:

Okay! even that person needs to be counseled or talked to about the condition, educated properly about the condition, and the line of treatment because without that the person can be depressed.

-Nurse, male, 23years

*There is a lot of guidance, a lot of counseling, lots of teaching, a lot of advice and training that you have to give to them, and it must be precise and accurate information so that it is easy for them to understand, and apply it as well*.

-Physiotherapist, male, 30 years

Moreover, for an effective therapeutic communication with patients, the health professionals indicated that as part of their practices they must communicate in a common language to enable the patient to freely express themselves. For instance, a female nurse aged 27years said *“Ok, first of all, I make sure they understand the language”.* Others also said;

*So, I try my best to interact with them in a common language that they understand, especially if I understand that language.*

-Physiotherapist, Female,28years

*So, during the assessment language is one thing that I consider for effective communication. I have to encode whatever message I need to give them for instance, how they have to perform their exercises. The number of repetitions they need to do I have to encode in a way that they can understand better. That’s really an aspect of the treatments.*

-Nurse, Male,28years

### Barriers to therapeutic communication

Barriers to therapeutic communication were reported in both quantitative and qualitative method. Table 5 present the quantitative results on barriers to therapeutic communication between health workers and patients. From the study, it was noted that the majority of the respondent’s 59.6% reported language as a major barrier to patient-health worker communication. Also, the result revealed that 55.2% of the patients do not clearly understand the information the health professionals communicate to them during their interaction. It was found that 744% of the patients who reported healthcare cost to be a challenge have poor communication with their healthcare practitioner. On the other hand, the difference in age, difference in gender, and severity of health conditions do not limit the means of communication between the health practitioner and patients.

**Table 5:** Barriers to therapeutic communication of CNCDs patients at HTH

Variables	Frequency (250)	Percent (%)
**Difference in gender makes it difficult for me to express myself**		
Disagree	208	83.2
Agree	42	18.8
**Age difference between the health professional and I make it a challenge to communicate**		
Disagree	193	77.2
Agree	57	22.8
**Language makes communication a problem**		
Disagree	101	40.4
Agree	149	59.6
**Time allocated for consultation is not enough in other to express myself better**		
Disagree	192	76.8
Agree	58	23.2
**Lack confidence makes it difficult to express myself**		
Disagree	228	91.2
Agree	22	8.8
**I do not feel like sharing my health conditions to anyone**		
Disagree	230	92.0
Agree	20	8.0
**I sometimes do not understand what the health professionals say sometimes**		
Disagree	112	44.8
Agree	138	55.2
**Previously had a bad experience with a health professional and that makes it difficult for me to express myself**		
Disagree	167	66.8
Agree	83	33.2
**Because there are more patients to attend to, the health professional usually rushes conversations in other to attend to the next person**		
Disagree	158	63.2
Agree	92	36.8
**When there are other duties for the health professional to perform, communication is mostly snappy**		
Disagree	166	66.4
Agree	84	33.6
**The health professional usually hesitates to open up to me making it difficult to get a clear understanding**		
Disagree	217	86.8
Agree	33	13.2
**The stress and pain I go through make it difficult for me to communicate**		
Disagree	181	72.4
Agree	69	27.6
**During a conversation with the health professional, interruptions from other work colleagues and calls make communication ineffective**		
Disagree	162	64.8
Agree	88	35.2
**Noise and disturbances around the consultation area do not enable concentration during communication**		
Disagree	196	78.4
Agree	54	21.6
**The consultation room is comfortable and well-ventilated, ensuring a pleasant environment**		
Disagree	12	4.80
Agree	238	95.2
**Severity of my case draws a lot of concentration at me and that makes me uncomfortable**		
Disagree	228	91.2
Agree	22	8.8
**Healthcare costs constrain my ability to freely express myself**		
Disagree	64	25.6
Agree	186	74.4

In agreement with the quantitative approach, below shows the qualitative results on barriers that inhibit effective therapeutic communication between health professionals and patients with CNCDs. From the perspective of the health personnel, two sub-themes were realized. The health-system related which had a total of six codes and the patient-related factors which had five codes. This is what they had to say respectively:

Some of them could be the language differences. It’s not every client that is well educated to be able to speak English or other local dialects. Some of them since they’ve been limited to a certain setting it becomes a challenge if you can’t really express yourself to the patient.

-Physiotherapist, male, 30years

*First one is the state of the patients because the consciousness of the patients mostly from acute set up to some months most of them have speech problems, some are not receptive, they don’t get to understand somehow virtually disoriented and that can be a barrier to communication”……“And then network reservations; some patients come from very far those areas telecom networks are not so good and because sometimes you wish to reach to the patient and do some follow-ups and get feedback but here is the case your call doesn’t go through*.

- physiotherapist, male, 30years

### Facilitators of therapeutic communication

The Table 6 presents the results on the facilitators of therapeutic communication. First, there is a clear indication the families of patients provide various assistance to their relations in other for them to get adequate healthcare and this sense of belonging is known to aid the patient recovery process. Assistance with taking medications scored (77.2%,) which indicates that having a support system usually facilitates well-being. Another good facilitation of communication is the availability of trust. More than two-thirds of the participants agreed that the ability of the health worker to create a good and friendly atmosphere, give recommendations in other to improve health, and also the health professional ability to communicate in plain language to the understanding of their patients all together contribute to facilitating a very good therapeutic communication.

**Table 6:** Facilitators of therapeutic communication for CNCD patients at HTH

Variables	Frequency [250]	Percent [%]
**My family provides me with the necessary help I need in other to get to the facility**		
Disagree	62	24.8
Agree	188	75.2
**My family assists me in taking my medications**		
Disagree	57	22.8
Agree	193	77.2
**The health professional creates a good atmosphere for communication since we have good relations**		
Disagree	17	6.8
Agree	233	93.2
**I have trust in my healthcare provider and I feel motivated to follow their advice**		
Disagree	24	9.6
Agree	226	90.4
**Information is given on recommendations in other to improve my health**		
Disagree	21	8.4
Agree	229	91.6
**The health professional creates a friendly atmosphere with enables me to communicate**		
Disagree	17	6.8
Agree	233	93.2
**The health professional communicates in plain language and in a way, I can understand whatever is communicated**		
Disagree	20	8.0
Agree	230	92.0

### Association between -demographic characteristics and therapeutic communication practices

Table 7 Presents the result of the logistic regression analysis conducted for effective practices and socio-demographic characteristics that were significant in the chi-square analysis. In male respondents (aOR = 0.92,95%Cl = 0.46–1.84, *p* = 0.817**)** were 92% less likely to practice effective therapeutic communication than the female respondents. However, the respondent who recorded having civil service as their predominant occupation were 5.16 times (aOR = 5.16,95%Cl = 1.75–15.17, *p* = 0.003) more likely to practice effective therapeutic communication compared to artisans.

**Table 7:** Bivariate association between patients’ socio-demographic and the predictors of therapeutic communication practices at HTH

Variables	Practices regarding therapeutic communication		Chi-square value (X^2^) P- value	COR (95% CI) [*P*-value]	AOR (95% CI) [*P*-value]
	Bad practices n (%)	Good practices n (%)			
**Age**			7.804(0.099)		
20–29	4(30.8)	9(69.2)			
30–39	7(21.9)	25(78.1)		1.59(0.37–6.74),0.531	
40–49	17(44.7)	21(55.3)		0.55(0.14–2.09),0.381	
50–59	37(49.3)	38(50.7)		0.46(0.13–1.61),0.223	
60+	37(40.2)	55(59.8)		0.66(0.19–2.30),0.526	
**Sex**			5.708(0.017)		
Female	45(33.8)	88(66.2)			
Male	57(48.7)	60(51.3)		**0.54(0.32–0.89),0.017**	**0.92(0.46–1.84),0.817**
**Level of education**			5.477(0.242)		
No formal education	14(50.0)	14(50.0)		0.5(0.19–1.31),0.161	
Primary	12(54.6)	10(45.5)		0.42(0.15–1.18),0.100	
JHS	15(33.33)	30(66.7)			
Secondary	29(45.3)	35(54.7)		0.60(0.27–1.33),0.211	
Tertiary	32(35.2)	59(64.8)		0.92(0.43–1.96),0.833	
**Main occupation**			14.979(0.005)		
Civil servant	20(27.8)	52(72.2)		**2.23(1.05–4.72),0.036**	**4.99(1.75–14.22),0.003**
Farmer	17(60.7)	11(39.3)		0.55(0.22–1.41),0.216	
Trader	14(30.4)	32(69.6)		1.96(0.85–4.50),0.113	
Unemployed	27(51.9)	25(49.1)		0.79(0.37–1.71),0.556	
Artisan	24(46.2)	28(53.9)			
**Religion**			12.418(0.002)		
African Traditional	8(100.0)	0(0.0)		1(empty)	1(empty)
Christianity	90(38.5)	144(61.5)		1.6(0.39–6.56),0.65	
Islam	4(50.0)	4(50.0)		-	-
**Marital status**			4.800(0.187)		
Never married	9(36.0)	16(64.0)		2.46(0.83–7.28),0.103	
Married	66(39.5)	101(60.5)		2.12(0.97–4.61),0.059	
Widowed	9(33.3)	18(66.7)			
Widowed	9(33.3)	18(66.7)		2.77(0.95–8.09),0.063	
**Ethnicity**			7.955(0.093)		
Akan	18(51.4)	17(48.6)			
Ewe	60(36.6)	104(63.4)		1.84(0.88–3.83),0.105	
Ga/Dangme	3(27.3)	8(72.7)		2.82(0.64–12.44),0.170	
Guan	19(57.6)	14(42.4)		0.78(0.29–2.20),0.611	
Mole-Dagbani	2(28.6)	5(71.4)		2.65(0.45–15.52),0.281	
**Persons in Household**			0.7869(0.375)		
1–5	92(41.8)	128(58.2)		1.44(0.64–3.22),0.377	
6–10	10(33.3)	20(66.7)			
**Health financing**			5.098(0.078)		
Self	59(46.5)	68(53.5)			
NHIS	13(27.7)	34(72.3)		0.75(0.42–1.34),0.332	
Family support	30(39.5)	46(60.5)		1.71(0.78–3.75),0.184	
**Diagnosed CNCD**			1.93(0.588)		
Diabetes	22(35.5)	40(64.5)			
Hypertension	26(38.2)	42(61.8)		0.89(0.44–1.81),0.745	
Stroke	27(43.6)	35(56.5)		0.71(0.35–1.47),0.359	
Comorbid(Diabetes, Hypertension)	17(31.5)	37(68.5)		1.19(0.55–2.59),0.649	

### Discussion

In this study we explored therapeutic communication between health professionals and patients with CNCDs at the HTH using a mixed method approach. We found that 59.2% of patients had good therapeutic communication with the health professionals. This findings could be attributed to a strong rapport built between patients and their health professional during counselling and education session which makes the patient feel relaxed in any further therapeutic interaction with the health professional. This is consistent with previous study which suggested that greetings, calling of patients with polite titles, the use of excuses by physicians before exiting, and the encouragement, motivation, and prayers all from physicians serve as practices for therapeutic communication [20, 21]. This implies that a warm welcome and kindness expressed by healthcare professionals serves as a gateway to good communication practices between patients and the health professionals. Regarding the socio-demographic predictors of therapeutic communication, we found male patients were less likely to practice effective good therapeutic communication compared with females. This finding is congruent with a study that have shown that female patients talk more about their feelings than men [21]. This observation in our study could be ascribed to the fact that females are more concern about their health therefore have higher tendency of discussing any health issues with their health professionals.

We found that patients’ trust in their healthcare professional serve as a facilitator for good therapeutic communication. This finding is consistent with a study conducted in Singapore by Shirazian et al. [22], which found that patients who trusted their health professionals felt more motivated to follow their advice and recommendations. Spyropoulos [23] highlighted that good communication between health professionals and patient relies on trust in the chosen health professional. Once this confidence is present, the patient must be completely truthful, and must also be compliant with his recommendations and instructions. This implies that trust plays an imperative role in establishing good therapeutic communication between patients and the health professionals.

Our study also found that the family’s assistance of patients during their interaction with the healthcare providers facilitates good therapeutic communication. This could be explained by the fact that majority of the chronic patients are elderly people who might have some difficulties with communication such as understanding English or other native languages spoken by healthcare professionals, therefore relatives accompanying patients becomes immediate interpreters that facilitate communication. This finding is congruent with a study conducted in Cambodia [24] which identified, family members as facilitators since they provide instrumental communication support between patient and their healthcare providers.

The study found that one major barrier to good therapeutic communication was language difference. This was a major issue for the health professionals who could not speak the native language (Ewe) spoken by the majority of patients. This is consistent with a study by Amoah et al. [15] which outlined that language is a major barrier to therapeutic communication. Similarly, in many studies, the health professional’s unfamiliarity with the patient’s colloquial language has been mentioned as a communication barrier [14–15, 25–28]. Due to this language problem, patients who cannot speak English and do not have interpreters to assist them find it difficult to communicate with their healthcare providers.

We discovered that healthcare providers using medical terminology during patient interactions impedes effective therapeutic communication. This may be because many patients are elderly, illiterate, or unfamiliar with medical terminology, making it difficult for them to understand healthcare professionals. This observation is consistent with a previous study by Qenam et al. [29] where they found that simplification is necessary to make patients understand what they have been told.

Limitations of the study.

Despite the relevance of the findings in this study, it is important to indicate the potential limitations inherent in the study. Because the study was cross-sectional, it could only measure associative rather than causal effects. The use of in-depth interviews for the data collection could have introduced response bias on the part of the participants. The use of purposive sampling for the qualitative aspect of the study could have introduced the possibility of selection bias on the part of the data collectors. Nonetheless, the strengths of the findings are rooted in the study design, data collected using standard methodologies, and relatively large sample that could facilitate generalization of the findings to larger population from which the sample was withdrawn from.

## Conclusion

The study revealed that there was good therapeutic communication between health professionals and patients with CNCDs. Furthermore, it is essential for healthcare professionals to establish trust with patients, as patient compliance with treatment relies on this trust. Patients should also receive support from their friends and family to foster a sense of belonging. Creating an open and welcoming atmosphere for patient communication is crucial.

Nevertheless, it was also identified that some of the barriers identified included language and the challenge of simplifying medical terminology for patients better understanding. These could lead to dissatisfaction with care, misdiagnosis, and noncompliance with the treatment regimen.

There is a need for Ghana Health Service and other relevant stakeholders to help improve patient quality of life through effective therapeutic communication. For instance, they should deploy healthcare professionals who are multilingual and conversant with the native language to the various health facilities. Also, the hospital administration should organize regular in-service training for their staff on the use of common medical terminologies to be used with patients.

## Data Availability

All relevant data are within the manuscript and its Supporting Information files. Any further requests regarding the data used for this study could be made through the corresponding author.

## Abbreviations

CNCDs: Chronic non-communicable diseases
SDG: Sustainable development goal
PCC: Patient-centered care
HTH: Ho Teaching Hospital
COREQ: Consolidated criteria for reporting qualitative research
STROBE: Strengthening the reporting of observational studies in Epidemiology
